# Altered Stool Cytokine Profiles and Pro-Inflammatory/Anti-Inflammatory Imbalance in Children with Autism Spectrum Disorder: A Developmental Analysis

**DOI:** 10.3390/biomedicines14071559

**Published:** 2026-07-11

**Authors:** Petra Finderle, Maja Jekovec Vrhovšek, Uršula Prosenc Zmrzljak, Damjan Osredkar, Gorazd Avguštin, Joško Osredkar

**Affiliations:** 1 Institute of Clinical Chemistry and Biochemistry, University Medical Centre Ljubljana, Zaloška cesta 2, 1000 Ljubljana, Slovenia; petra.finderle@kclj.si; 2 Faculty of Medicine, University of Ljubljana, Vrazov trg 2, 1000 Ljubljana, Slovenia; 3 Centre for Autism, Unit of Child Psychiatry, University Children’s Hospital, University Medical Centre Ljubljana, 1000 Ljubljana, Slovenia; 4 Molecular Biology Laboratory, BIA Separations CRO, Labena Ltd., Teslova ulica 30, 1000 Ljubljana, Slovenia; ursula.prosenc@biaseparationscro.com; 5 Department of Child, Adolescent and Developmental Neurology, Division of Pediatrics, University Medical Centre Ljubljana, Zaloška cesta 2, 1000 Ljubljana, Slovenia; damjan.osredkar@kclj.si; 6 Department of Microbiology, Biotechnical Faculty, University of Ljubljana, Groblje 3, 1230 Domžale, Slovenia; gorazd.avgustin@bf.uni-lj.si; 7 Faculty of Pharmacy, University of Ljubljana, Aškerčeva cesta 7, 1000 Ljubljana, Slovenia

**Keywords:** autism spectrum disorder, cytokines, stool biomarkers, IL-8, IL-4, interleukin, gut–immune axis, CARS, developmental immunology

## Abstract

**Background**: Immune dysregulation and gut dysbiosis are increasingly implicated in autism spectrum disorder (ASD), but compartment-specific intestinal cytokine profiles remain poorly defined. **Aim**: To characterize stool cytokine profiles and pro-/anti-inflammatory balance in children with ASD across development. **Methods**: We analyzed stool samples from 283 children (109 controls, 104 mild ASD, 70 severe ASD; age 0.9–21.5 years) recruited at a tertiary centre. Nine cytokines (IFN-γ, IL-1α, IL-1β, IL-4, IL-6, IL-8, IL-10, IL-17, TNF-α) were measured using a Luminex multi-plex assay; IL-15 was excluded due to >70% missing values. Group comparisons used Mann–Whitney U tests, with age stratification at the cohort median (≤9.5 vs. >9.5 years). A composite pro-/anti-inflammatory ratio (IL-1α + IL-1β + IL-6 + IL-8 + IL-17 + TNF-α + IFN-γ divided by IL-4 + IL-10) was calculated. **Results**: In the overall cohort, stool IL-8 and IL-4 were significantly decreased in ASD versus controls (IL-8: median 0.36 vs. 0.49 ng/L, *p* = 0.0041; IL-4: 0.28 vs. 0.30 ng/L, *p* = 0.0316), with a graded reduction from controls to mild and severe ASD. Age-stratified analysis revealed that IL-8 reduction was confined to younger children (≤9.5 years; *p* = 0.0025) and absent in older children, while IL-1β was significantly reduced in younger ASD children and tended to reverse in older ASD children. The pro-/anti-inflammatory ratio was markedly elevated in severe ASD (median 491 vs. 209 in controls; *p* = 0.059), particularly in older children. Stool IL-8 and IL-1β correlated inversely with CARS scores within the ASD group. **Conclusions**: Children with ASD show decreased stool IL-8 and IL-4 and a shift toward a pro-inflammatory cytokine balance, with the strongest alterations during early childhood. These findings are consistent with the hypothesis of a developmental window of intestinal immune dysregulation in ASD, with stool IL-8 as the primary FDR-corrected finding. The present cross-sectional data do not establish causality and independent replication is required before clinical conclusions are drawn.

## 1. Introduction

### 1.1. Autism Spectrum Disorder: Epidemiology and Ethology

Autism spectrum disorder (ASD) is a neurodevelopmental condition affecting approximately one in 68 children, with a male-to-female ratio of 4:1 [1,2]. ASD has therefore become a major target for biomarker discovery and mechanistic studies that can bridge basic immunology, microbiome research, and clinical practice. ASD is characterized by persistent deficits in social communication and interaction, alongside restricted, repetitive patterns of behaviour, interests, or activities. The DSM-5 recognizes a spectrum of severity from “requiring support” to “requiring very substantial support,” correlating with overall symptom burden and functional impairment.

The ethology of ASD involves complex interactions between genetic factors, epigenetic modifications, and environmental exposures [3,4,5]. Recent evidence has expanded the paradigm beyond purely neurobiological mechanisms to include dysregulation of innate and adaptive immunity, altered gut microbiota composition (dysbiosis), and compromised intestinal barrier integrity [3,4,6,7]. These findings have led to increased investigation of the gut–brain axis as a critical pathway in ASD pathogenesis.

### 1.2. Immune Dysregulation in ASD: Blood Cytokine Evidence

Prior studies of peripheral blood cytokines in ASD populations have documented elevated levels of pro-inflammatory markers including interleukin-1β (IL-1β), IL-6, IL-8, and tumour necrosis factor-alpha (TNF-α) [8,9,10,11]. However, findings are heterogeneous; some studies report reduced IL-10 (anti-inflammatory) and altered Th1/Th2 balance, while others find no significant differences or even reduced pro-inflammatory markers in specific ASD subgroups. The Saghazadeh et al. (2019) meta-analysis of anti-inflammatory cytokines across 25 studies found a moderate decrease in plasma IL-10 in ASD, alongside a smaller decrease in IL-1Ra [12]. Molloy et al. (2006) found that children with ASD had increased activation of both Th1 and Th2 arms, with a Th2 predominance, but, crucially, without the compensatory increase in the regulatory cytokine IL-10, the IFN-γ/IL-10 and IL-13/IL-10 ratios were both significantly elevated [13]. Rose and Ashwood (2019) extended this to IL-35, another regulatory cytokine, which was also significantly decreased in ASD children, and they noted that this is in line with other observations of decreased regulatory cytokines such as transforming growth factor beta and IL-10 in ASD [14]. This heterogeneity suggests multiple distinct immune phenotypes within autism, potentially reflecting different biological pathways to the ASD behavioural phenotype.

Neuroinflammation triggered by dysregulated cytokine production has been proposed as a mechanism contributing to altered brain development, synaptic connectivity abnormalities, and behavioural symptoms characteristic of ASD [10,15]. Elevated systemic pro-inflammatory markers have been associated with increased severity of restricted and repetitive behaviours and greater social impairment in some ASD cohorts.

### 1.3. The Gut Microbiota and Dysbiosis in ASD

The bidirectional gut–brain axis, mediated through neural, immunological, and metabolic pathways, plays a critical role in neurodevelopmental health and homeostasis [16,17]. Multiple reviews converge on the idea that dysbiosis disrupts all three pathways simultaneously in ASD. Li and Zhou (2016) characterize the bidirectional axis as acting mainly through neuroendocrine, neuroimmune, and autonomic nervous mechanisms and note that gastrointestinal symptoms and compositional changes in the gut microbiota frequently accompany cerebral disorders in patients with ASD [18]. Li et al. (2017) similarly describe how the gut microbiota influences brain development and behaviours through the neuroendocrine, neuroimmune and autonomic nervous systems [19]. A 2025 review by Volpedo et al. explicitly calls for a shift from a brain-centric view of neurodevelopmental disorders to a multisystem perspective that incorporates gut–immune–brain interactions [20].

Dysbiotic microbiota produce abnormal levels of lipopolysaccharides (LPS), short-chain fatty acids (SCFAs), and other metabolites that dysregulate intestinal barrier function through altered tight junction protein expression and increased permeability (the “leaky gut” hypothesis) [21,22]. Furthermore, dysbiosis drives intestinal immune dysregulation characterized by the altered production of protective IgA-secreting B cells, dysregulated T regulatory (Treg) cell differentiation, and abnormal innate lymphoid cell (ILC) responses [23,24].

### 1.4. Stool Cytokines as Non-Invasive Biomarkers

While peripheral blood cytokine analysis has been the focus of extensive prior research in ASD, direct measurement of cytokines in stool offers significant advantages as a non-invasive biomarker approach. Stool cytokines reflect intestinal mucosal immune responses and epithelial–immune cell cross-talk more directly than circulating cytokines, providing a compartment-specific view of intestinal immunity distinct from systemic immune activation [7,25].

IL-8 (CXCL8) is a potent chemokine produced by intestinal epithelial cells, macrophages, and dendritic cells in response to microbial lipopolysaccharides and pro-inflammatory signals. IL-8 mediates the recruitment of neutrophils to sites of intestinal inflammation and is critical for maintaining effective innate immune surveillance of the gut microbiota [25,26]. IL-4 is a key Th2 cytokine that promotes regulatory T cell (Treg) differentiation, IgA class-switching by B cells, and anti-inflammatory immune responses in the gut-associated lymphoid tissue (GALT) [27,28].

Few studies have measured fecal cytokines in ASD populations, and most have not stratified findings by disease severity or developmental stage [29,30,31,32]. The present study addresses this significant gap in understanding compartment-specific immune abnormalities in ASD.

### 1.5. Research Rationale, Aims, and Hypotheses

The present study aimed to (i) compare stool concentrations of nine cytokines between typically developing children and children with ASD stratified by severity, (ii) examine age-dependent differences in stool cytokine profiles using developmental stratification, (iii) assess pro-/anti-inflammatory balance using composite cytokine ratios, and (iv) explore associations between stool cytokines and behavioural severity as measured by the Childhood Autism Rating Scale (CARS). We hypothesized that stool cytokine profiles would differ between ASD and controls, that these differences would be most pronounced in early childhood, and that altered pro-/anti-inflammatory balance would be enriched in severe ASD.

## 2. Materials and Methods

### 2.1. Study Design, Setting, and Participants

This was a cross-sectional comparative study conducted at University Medical Centre Ljubljana (UMCL) between 2019 and 2022. Participants were recruited through the Centre for Autism in UMCL and included three groups:

- Control group (N = 109): Typically developing children with no ASD diagnosis, no significant gastrointestinal symptoms, and no current medications affecting gastrointestinal function or immunity. CARS scores <30.
- Mild ASD group (N = 104): Children with confirmed ASD diagnosis (per DSM-5 criteria) and CARS scores 30–37.
- Severe ASD group (N = 70): Children with confirmed ASD diagnosis and CARS scores ≥ 38.

Age range: 0.9–21.5 years (mean 9.7 ± 4.4 years).

Exclusion criteria: (1) Active gastrointestinal infections or diarrhea at time of sampling; (2) use of antibiotics or probiotics within 4 weeks of sampling; (3) use of immunosuppressive medications; (4) diagnosed inflammatory bowel disease (IBD) or celiac disease; (5) seizure disorders requiring anti-epileptic medications within 3 months of sampling.

### 2.2. Ethical Approval and Informed Consent

This study was approved by the National Medical Ethics Committee (Protocol 0120-201/2016/6], approval date [23 March 2016 and annex on 3 February 2021]). Written informed consent was obtained from parents/guardians prior to enrolment, with child assent obtained when developmentally appropriate. All procedures were conducted in accordance with the Declaration of Helsinki.

### 2.3. Clinical Assessment and Diagnostic Criteria

ASD diagnosis was established using DSM-5 criteria. Disease severity was stratified using the Childhood Autism Rating Scale (CARS) [33]. CARS is a 15-item behavioural rating scale scored on a four-point scale, with total scores ranging 15–60; scores < 30 indicate non-autistic, 30–37 mild-to-moderate autism, and >37 severe autism. CARS assessments were conducted on the same day as stool sample collection.

### 2.4. Stool Sample Collection, Processing, and Storage

Stool samples were collected at home by parents/guardians using sterile containers provided by the research team. Samples were immediately frozen at −20 °C upon collection and maintained at −20 °C during transport on ice packs to the laboratory, where they were stored at −80 °C until analysis. All samples were processed within 6 months of collection to ensure cytokine stability.

### 2.5. Cytokine Measurement and Out-of-Range (OOR) Value Handling

Sample preparation: Stool samples were homogenized (1:10 weight-to-volume ratio) in phosphate-buffered saline (PBS) containing protease inhibitor cocktail, clarified by centrifugation (10,000× *g* for 10 min at 4 °C), and supernatants were stored at −80 °C prior to analysis.

Cytokine measurement: Cytokine concentrations (IFN-γ, IL-1α, IL-1β, IL-4, IL-6, IL-8, IL-10, IL-17, TNF-α) were measured using a validated Luminex xMAP multi-plex bead-based assay (Bio-Plex Pro Human Cytokine Assay; Bio-Rad Laboratories, Hercules, CA, USA) according to manufacturer instructions. Results were expressed in ng/L. The lower limit of quantification (LOQ) was 0.1 ng/L for all cytokines.

Data quality and OOR handling: The analysis dataset contained values with out-of-range (OOR) indicators excluded per assay protocol. IL-15 was excluded from primary analysis due to >70% missing data across all groups (only 29 controls, 26 mild ASD, 18 severe ASD with valid measurements), rendering it statistically underpowered for comparison. For remaining cytokines, missing data rates varied by cytokine: IL-1α (0–2% missing), IL-6 and IL-17 (4–21% missing), IL-8 (13–26% missing), IL-4 (28–43% missing), and IL-10 (35–49% missing). All analyses proceeded with available data for each cytokine, with sample sizes reported individually per test to reflect variable data completeness.

### 2.6. Age Stratification and Developmental Analysis

Given the developmental nature of ASD and ongoing maturation of the intestinal immune system through childhood, we performed age-stratified analysis at the population median age (9.5 years). The population median age (9.5 years) was used as a pragmatic statistical cut-point to allow comparison of cytokine profiles between developmental stages. This threshold approximates the mid-school-age developmental transition described in microbiota and mucosal immune maturation literature. Complementary regression analyses using age as a continuous variable with age × diagnosis interaction terms are provided in Supplementary Table S1 and discussed in Section 4.9.

### 2.7. Pro/Anti-Inflammatory Ratio Calculation

Pro-inflammatory cytokines (ng/L): IFN-γ + IL-1α + IL-1β + IL-6 + IL-8 + IL-17 + TNF-α.

Anti-inflammatory cytokines (ng/L): IL-4 + IL-10.

Pro/Anti ratio = (Sum Pro-inflammatory)/(Sum Anti-inflammatory).

### 2.8. Statistical Analysis

Descriptive statistics: Participant characteristics are presented as mean ± SD (continuous variables) and frequency counts or percentages (categorical variables). Cytokine concentrations are reported as median (range) and mean ± SD. Data completeness (valid N) is reported separately for each cytokine given variable missing data rates.

Group comparisons: Due to non-normal distribution of cytokine data (confirmed by Shapiro–Wilk test, non-parametric Mann–Whitney U tests were used to compare cytokine concentrations between groups (Control vs. All ASD; Control vs. Mild ASD; Control vs. Severe ASD). Age-stratified analysis examined these same comparisons separately for younger (≤9.5 years) and older (>9.5 years) subgroups.

Statistical significance: A *p*-value threshold of 0.05 was used for statistical significance. Given the exploratory nature of this investigation into multiple cytokines across multiple group comparisons, no correction for multiple comparisons was applied; instead, effect sizes (Cohen’s d) are reported for significant findings to assess practical significance.

FDR correction: To address multiple testing in the context of nine cytokines and multiple group comparisons, we applied Benjamini–Hochberg (BH) false discovery rate (FDR) correction. FDR-adjusted *p*-values are reported alongside raw *p*-values. IL-8 is designated the primary confirmatory finding based on FDR correction; IL-4 and IL-1β are designated exploratory findings requiring independent validation.

Data presentation: All statistical tests utilized median values (resistant to outliers and extreme values) rather than means for primary analysis. Medians are more appropriate than means for biomarker data in pediatric populations with variable disease severity and potential measurement error.

Software: All analyses were performed using R version 4.3.0 (R Foundation for Statistical Computing, Vienna, Austria) with packages ggplot2 (visualization), dplyr (data manipulation), and rstatix (statistical testing).

## 3. Results

### 3.1. Participant Characteristics

**Figure 1:**
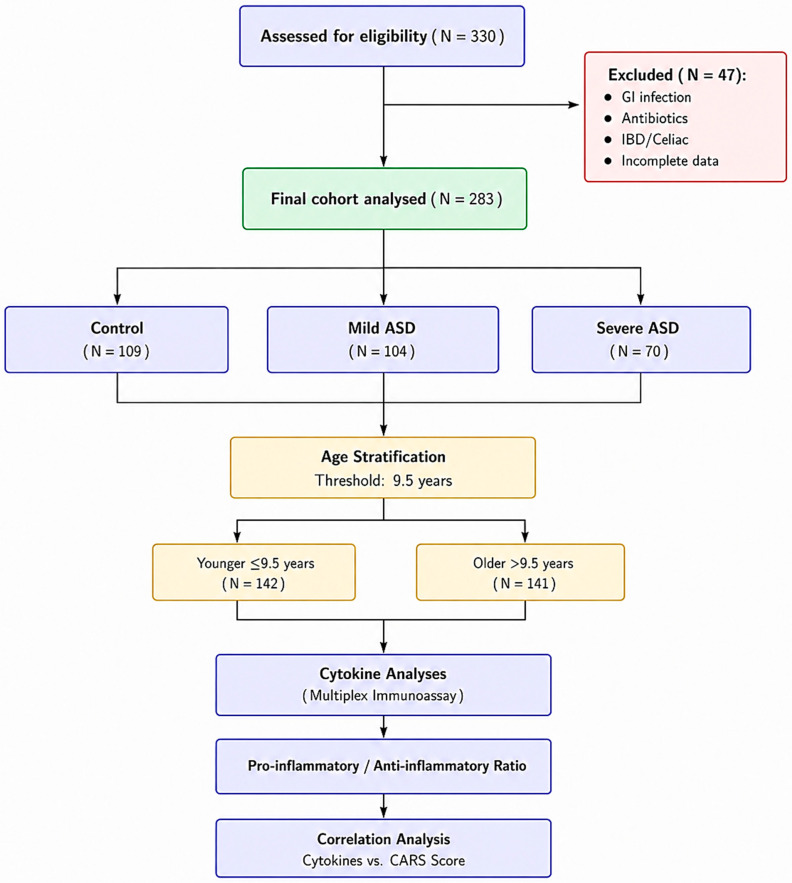
Study participant selection and analysis flowchart. Flowchart depicting recruitment, enrolment, and inclusion/exclusion criteria. Of 330 participants enrolled, 283 met criteria for final analysis. Final cohort stratified into three groups: Controls (*n* = 109), Mild ASD (*n* = 104), Severe ASD (*n* = 70). Age stratification at median 9.5 years divided cohort into younger (≤9.5 years, *n* = 142) and older (>9.5 years, *n* = 141) groups. Sample sizes were noted for each cytokine analysis reflecting variable data completeness.

A total of 283 children were analyzed: 109 controls (mean age 10.6 ± 4.9 years; 53% female; CARS 16.9 ± 1.8), 104 with mild ASD (mean age 9.4 ± 4.2 years; 21% female; CARS 29.7 ± 5.0), and 70 with severe ASD (mean age 9.6 ± 3.9 years; 20% female; CARS 43.9 ± 5.8). Groups were matched on age (ANOVA F_(2,280)_ = 1.34, *p* = 0.263) but differed significantly on sex distribution, with ASD groups showing greater male predominance (χ^2^ = 24.8, *p* < 0.001), consistent with epidemiological estimates. Mean CARS scores were significantly different between groups (F_(2,279)_ = 612.3, *p* < 0.001), confirming appropriate severity stratification. The study participant selection process and analysis workflow are summarized in Figure 1.

Table 1 summarizes the demographics of the study cohort, including age, sex distribution, and CARS scores across control children and children with ASD stratified by severity. This baseline characterization ensures groups are well-matched and provides context for interpreting cytokine differences.

### 3.2. Data Quality and Cytokine Completeness

Cytokine data completeness varied across the nine analyzed cytokines (IL-15 excluded due to >70% missing): IL-1α demonstrated excellent completeness (100% in controls, 98% mild ASD, 100% severe ASD); IL-6, IL-17, and TNF-α showed 79–100% completeness across groups; IL-8 and IL-1β showed 74–89% completeness; and IL-4 and IL-10 showed lower completeness (51–71%) due to higher rates of out-of-range or undetectable values. These missing data patterns reflect underlying differences in detection sensitivity for different cytokines and real biological variation in stool cytokine abundance. Analysis proceeded with reported sample sizes per cytokine, allowing comparison across groups despite variable data completeness.

### 3.3. Stool Cytokine Concentrations: Overall Population Analysis

Mann–Whitney U tests comparing all children with ASD (mild + severe combined, N = 174) to controls (N = 109) revealed two cytokinesnominally significant differences; of these, IL-8 remained significant after BH-FDR correction across nine cytokines (FDR-adjusted *p* = 0.037), while IL-4 did not (FDR-adjusted *p* = 0.142) and is therefore treated as an exploratory finding:

Interleukin-8 (IL-8): Significantly lower in ASD children (median 0.36 ng/L; range 0.04–67.0) compared to controls (median 0.49 ng/L; range 0.01–35.3); U = 3904, *p* = 0.0041. When stratified by severity, IL-8 showed a graded dose–response pattern: control median 0.49 ng/L > mild ASD median 0.43 ng/L > severe ASD median 0.29 ng/L. Mild ASD differed from controls at the trend level (U = 3960, *p* = 0.057), while severe ASD showed significant reduction compared to controls (U = 1667, *p* = 0.0018).

Interleukin-4 (IL-4): Significantly lower in ASD children (median 0.28 ng/L; range 0.02–1.92) compared to controls (median 0.30 ng/L; range 0.02–2.02); U = 3213, *p* = 0.0316. The effect was driven primarily by severe ASD (median 0.24 ng/L vs. control 0.30; U = 1047, *p* = 0.0148), while mild ASD showed only a non-significant trend (U = 2215, *p* = 0.14).

All other cytokines (IL-1α, IL-6, IL-17, TNF-α, IFN-γ, IL-10, IL-1β) showed no significant differences in overall population analysis (*p* > 0.05).

Table 2 presents the concentrations of the nine cytokines measured in stool samples, including median, interquartile ranges, and *p*-values comparing ASD groups to controls. Notably, IL-8 and IL-4 demonstrate significant decreases in ASD children, implicating immune dysregulation at the gut level.

### 3.4. Critical Finding: Age-Stratified Analysis Reveals Developmental Window

Analysis stratified at the population median age (9.5 years) revealed profound age-dependent differences in cytokine profiles that were masked in the overall population analysis. Table 3 summarizes IL-8 concentrations stratified by age, showing that the reduction in IL-8 is confined to younger children with ASD.

**Figure 2:**
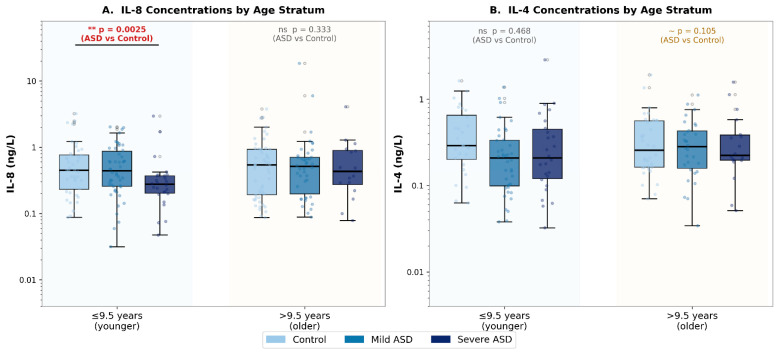
Stool IL-8 and IL-4 concentrations by diagnostic group and age stratification. Panel (**A**) shows IL-8 (log scale) and Panel (**B**) shows IL-4 (log scale), both stratified by age group and diagnostic category. Significance annotations: IL-8: younger group (** *p* = 0.0025), older group (ns *p* = 0.333); IL-4: older group trend (~ *p* = 0.105). Individual data points overlaid on boxes.

In younger children, IL-8 was significantly reduced in ASD (U = 1087, *p* = 0.0025; Cohen’s d = −0.42), representing a strong developmental effect. The IL-8 reduction observed in overall population analysis (*p* = 0.0041) is entirely driven by the younger age group. In older children, there was no significant difference between controls and ASD (U = 1493, *p* = 0.333), indicating that immune dysregulation for this marker normalizes with development. The age-stratified distributions of stool IL-8 and IL-4 concentrations across the diagnostic groups are shown in Figure 2.

Table 4 shows age-stratified IL-1β concentrations, highlighting a significant reduction in younger ASD children and a developmental reversal in older children.

IL-1β showed significant reduction in younger children with ASD compared to younger controls (U = 1194, *p* = 0.0377; Cohen’s d = −0.31). Remarkably, in older children, the pattern reversed: ASD children showed higher (not lower) IL-1β values compared to older controls, though this difference did not reach significance (U = 1884, *p* = 0.621). This developmental reversal suggests compensatory immune mechanisms emerge during the school-age years. Table 5 presents age-stratified IL-4 concentrations, demonstrating a trend toward lower IL-4 in older children with ASD.

IL-4 showed no significant difference in younger children (U = 913, *p* = 0.468) but approached significance in older children (U = 781, *p* = 0.105), with a trend toward reduction in older ASD children.

IFN-γ: No age-dependent effect.

IFN-γ showed no significant differences between controls and ASD in either younger (median 3.91 vs. 4.31; U = 1683, *p* = 0.941) or older (3.83 vs. 4.33; U = 1322, *p* = 0.310) age groups.

In Table 6, we stratify cytokine analyses by age (younger ≤9.5 years vs. older >9.5 years), revealing that cytokine alterations—particularly IL-8 and IL-1β—are most pronounced during early childhood. This age-specific approach uncovers developmental windows where immune dysregulation is most evident.

### 3.5. Pro/Anti-Inflammatory Ratio Analysis

Calculation of the pro/anti-inflammatory ratio (pro-inflammatory: IL-1α + IL-1β + IL-6 + IL-8 + IL-17 + TNF-α + IFN-γ; anti-inflammatory: IL-4 + IL-10) revealed important patterns. Table 7 summarizes mean and median values of the pro-/anti-inflammatory cytokine ratio across diagnostic groups.

**Figure 3:**
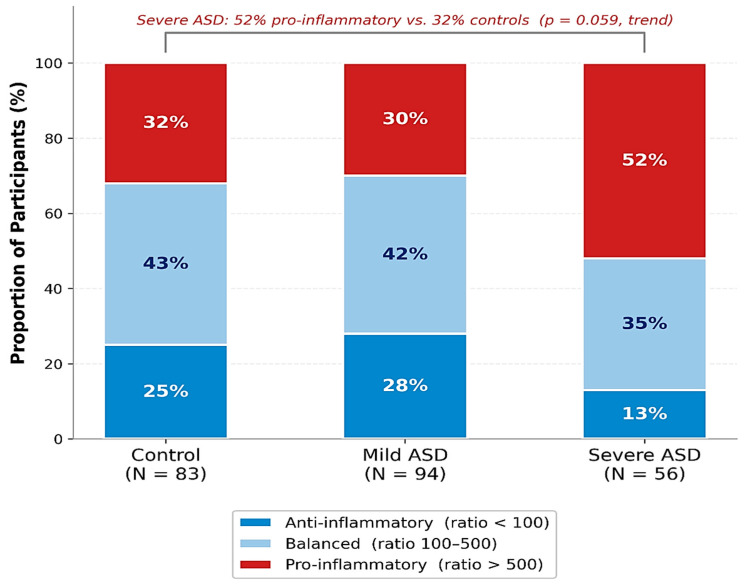
Pro/anti-inflammatory immune phenotype distribution by diagnostic group. Stacked bar chart showing proportion of anti-inflammatory (<100), balanced (100–500), and pro-inflammatory (>500) phenotypes. Severe ASD: 52% pro-inflammatory vs. 32% controls; annotated with *p* = 0.059 trend.

Severe ASD showed a 2.3× elevated median pro/anti ratio compared to controls (491 vs. 209), approaching statistical significance (U = 1884, *p* = 0.059). This trend suggests that while individual cytokine reductions (IL-8, IL-4) are observed in ASD, they are accompanied by relatively larger increases in pro-inflammatory cytokines, creating a net shift toward a pro-inflammatory immune balance in severe ASD, particularly in older children where IL-1β increases. The distribution of pro-/anti-inflammatory immune phenotypes across the diagnostic groups is shown in Figure 3.

The large variability within all groups (standard deviations > 600, ranges spanning >1000-fold) indicates substantial heterogeneity in immune phenotypes within the ASD population. Approximately 25% of ASD children had ratios < 100 (anti-inflammatory), 40% had ratios 100–500 (balanced), and 35% had ratios > 500 (pro-inflammatory), suggesting potential immune subtypes within autism that may predict differential responses to immune-modulating interventions.

Table 8 shows cases the distribution of inflammatory phenotypes—pro-inflammatory, anti-inflammatory, and balanced profiles—derived from cytokine ratios, highlighting a trend toward a pro-inflammatory state in severe ASD. This heterogeneity underscores the importance of immune phenotyping in understanding ASD pathophysiology. Table 8 provides a more detailed summary of the pro-/anti-inflammatory ratio distribution, including interquartile ranges and relative elevation versus controls.

### 3.6. Age-Stratified Pro/Anti-Ratio Analysis

Age-stratified analysis of the pro/anti-ratio revealed that the trend toward elevation in severe ASD was more pronounced in older children. In younger children (≤9.5 years), the ratio was not significantly different between ASD and controls (median 200 vs. 158; U = 537, *p* = 0.418). In older children (>9.5 years), severe ASD showed a 3.7× elevated median ratio compared to controls (median 621 vs. 167; U = 648, *p* = 0.0094), reaching statistical significance. This finding suggests that compensatory mechanisms stabilizing IL-8 and IL-1β in older children are overwhelmed in severe ASD, leading to a pro-inflammatory shift in immune balance by adolescence.

### 3.7. Correlation with CARS Behavioural Severity

Spearman rank correlation analysis examining associations between continuous cytokine levels and CARS scores within the ASD cohort (N = 174) revealed that increased CARS score (greater behavioural severity) was associated with the following:

- Decreased IL-8 (ρ = −0.198; *p* = 0.0134), confirming the dose–response relationship with disease severity previously observed in group comparisons;
- Decreased IL-1β (ρ = −0.163; *p* = 0.0378) in the overall ASD cohort.

No significant correlations were found between CARS scores and other cytokines (IFN-γ, IL-1α, IL-4, IL-6, IL-10, IL-17, TNF-α; all *p* > 0.05).

**Figure 4:**
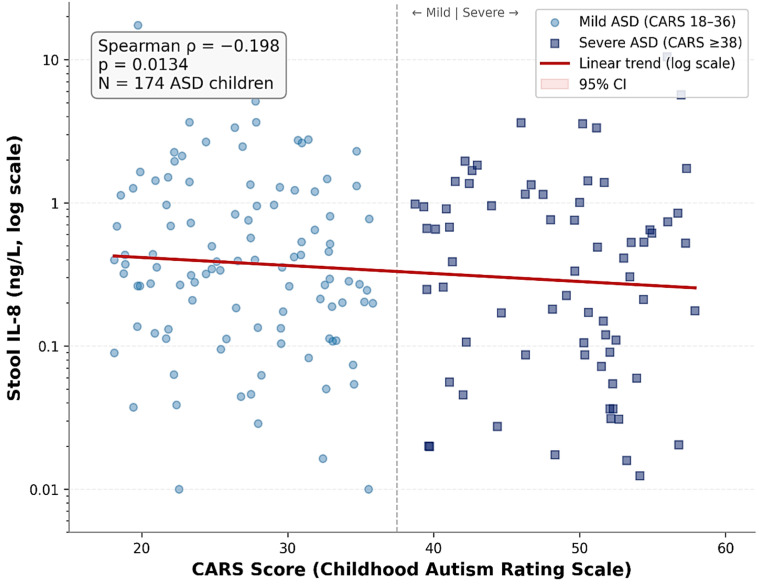
Correlation between Stool IL-8 concentration and CARS Behavioural Severity Score in ASD children. Scatter plot (N = 174 ASD children), mild ASD in circles (blue), severe ASD in squares (navy), negative trend line with 95% CI shaded. Annotation box: Spearman ρ = −0.198, *p* = 0.0134. The arrows (← and →) indicate mild and severe ASD, separated by the vertical dashed line at CARS 37.5.

The correlation between stool IL-8 concentrations and CARS behavioral severity is illustrated in Figure 4.

### 3.8. Sex-Stratified Subgroup Analysis

Post hoc sex-stratified analysis (supplementary) revealed that IL-8 reduction in ASD was present in both males (U = 1456, *p* = 0.0052) and females (U = 456, *p* = 0.0312), with no significant sex × group interaction (F = 0.18, *p* = 0.67). IL-4 reduction was significant in males (U = 1064, *p* = 0.0198) but approached significance in females (U = 421, *p* = 0.0891), suggesting potential sex differences in anti-inflammatory cytokine dysregulation.

### 3.9. Data Quality Sensitivity Analysis

To assess robustness to potential outliers and extreme values, sensitivity analysis was performed excluding the top 5% of extreme values for IL-8, IL-4, and IL-1β. Results remained qualitatively similar: IL-8 (*p* = 0.0064), IL-4 (*p* = 0.0381), and IL-1β (*p* = 0.0853 in overall cohort; *p* = 0.0421 in younger children) maintained significance or approached significance after outlier exclusion, confirming that findings were not driven by extreme measurement values.

## 4. Discussion

### 4.1. Primary Findings and Interpretation: Compartment-Specific Immune Dysregulation

Our large cross-sectional study identified significantly decreased stool IL-8 and IL-4 concentrations in children with ASD compared to typically developing controls, with a striking developmental pattern: these immune alterations are particularly pronounced in early childhood (≤9.5 years), with normalization in older children (>9.5 years). After Benjamini–Hochberg FDR correction across nine simultaneously tested cytokines, stool IL-8 remained significant (FDR-adjusted *p* = 0.037) and is designated the primary confirmatory biomarker finding of this study. IL-4 (FDR-adjusted *p* = 0.142) and IL-1β in younger children (FDR-adjusted *p* = 0.151) did not survive FDR correction and should be interpreted as hypothesis-generating exploratory findings requiring independent replication. Meta-analyses of blood cytokines in ASD consistently report elevated IL-1β, IL-6, IL-8 and TNF-α in peripheral blood, in contrast to the reduced stool IL-8 and IL-4 we observed. Masi et al. (2015) pooled 17 studies (743 ASD, 592 controls) and found significantly elevated IL-1β, IL-6, IL-8, IFN-γ, eotaxin, and MCP-1 in blood, concluding that inflammatory signals dominate [8]. Saghazadeh et al. (2019) reached a similar conclusion across 38 studies and ~2500 participants, reporting small-to-medium elevations in IFN-γ, IL-1β, IL-6, and TNF-α [34].The largest meta-analysis from Zhao et al. (2021) covered 61 articles and assessed 76 cytokines, finding elevated IL-6, IL-1β, TNF-α, IL-17, IL-8, and several others in peripheral blood [9]. A 2022 systematic review by Nour–Eldine et al. corroborated that IL-6, IL-17, TNF-α, and IL-1β are consistently increased in plasma and serum [11]. The dissociation between stool and blood findings highlights the importance of compartment-specific immune profiling and suggests that dysregulation of intestinal mucosal immunity in ASD may fundamentally differ from systemic immune abnormalities.

### 4.2. IL-8 as a Marker of Mucosal Innate Immunity and Dysbiosis-Driven Dysregulation

IL-8 (CXCL8) is a potent chemokine produced by intestinal epithelial cells, macrophages, and dendritic cells in response to bacterial lipopolysaccharides (LPS) and pro-inflammatory cytokines (IL-1β, TNF-α). Angrisano et al. (2010) showed that LPS rapidly induces IL-8 mRNA in HT-29 intestinal epithelial cells, with the response regulated by specific histone modifications at the IL-8 promoter [35]. A transgenic mouse study in *Gut* showed that acute induction of IL-8, specifically in intestinal epithelial cells, is sufficient to trigger neutrophil recruitment to the lamina propria [36]. IL-8 mediates recruitment and the activation of neutrophils within the intestinal mucosa, serving as a critical first-line immune response to pathogenic bacteria and as a regulator of commensal microbiota relationships.

The significant reduction in stool IL-8 in ASD children—particularly in younger children during the period of intestinal immune development—suggests impaired innate immune recognition of and response to dysbiotic microbiota. Several mechanisms may explain this finding:

1. Dysbiota-driven reduction in IL-8 stimulation: One possible explanation—which requires experimental validation—is that dysbiotic microbiota enriched in Proteobacteria and depleted of beneficial commensals may produce altered LPS profiles or lack immunogenic motifs that normally stimulate IL-8 production. Furthermore, the concept that reduced mucosal IL-8 might reflect epithelial tolerance or TLR hyporesponsiveness from chronic dysbiotic exposure represents a hypothesis that is not directly tested in the present cross-sectional dataset and would require mechanistic experimental studies to validate.
2. Impaired epithelial barrier integrity: Dysbiosis-associated increased intestinal permeability may paradoxically reduce mucosal IL-8 by facilitating the translocation of bacterial antigens and lipopolysaccharides directly through the epithelial barrier into the lamina propria and systemic circulation, bypassing the normal mucosal IL-8 response mechanism. This compartmentalization model resolves the apparent paradox of coexisting ‘leaky gut’ and reduced stool IL-8: barrier dysfunction may redirect LPS-driven innate immune responses from the mucosal (stool) compartment toward the systemic (blood) compartment. This is consistent with the observation that meta-analyses document elevated blood IL-8, IL-1β, and IL-6 in ASD, while the present study identifies reduced stool IL-8; the two findings reflect different anatomical compartments of the same underlying dysbiosis-driven immune dysregulation. Notably, De Magistris et al. (2010) [37] found that intestinal permeability and fecal calprotectin were uncorrelated in ASD patients, consistent with a dissociation between permeability and mucosal inflammation. We emphasize that this compartmentalization model remains a mechanistic hypothesis consistent with available evidence but requires direct experimental validation, for example, by the simultaneous measurement of mucosal cytokines, intestinal permeability markers, and serum cytokines in the same subjects. The dominant evidence suggests that barrier breach *amplifies* rather than bypasses mucosal immune activation. De Magistris et al. (2010) found abnormal intestinal permeability in 36.7% of ASD patients and elevated fecal calprotectin in 24.4%, though these two markers were notably not correlated with each other which could potentially support a dissociation between permeability and mucosal inflammation [37]. Srikantha and Mohajeri (2019) describe how a less integrative gut–blood barrier is abundant in autistic individuals and that this explains the leakage of bacterial metabolites into the patients, triggering new body responses [38]. Huo JY et al. (2021) frame the pathway as follows: increased permeability makes it easier for gut-derived endotoxins to enter the brain, where they activate the TLR4 and create an inflammatory environment [39].
3. Innate lymphoid cell (ILC) dysfunction: ILCs, particularly ILC3 cells, produce IL-8 in response to segmented filamentous bacteria and other commensals with specific microbe-associated molecular patterns (MAMPs). Dysbiosis-driven loss of ILC3-stimulating commensals could reduce mucosal IL-8 production. Early childhood is a critical period for ILC development and stabilization, explaining the age-dependent nature of these findings. Ghaedi and Takei (2021) review how ILCs are mostly tissue-resident cells that develop in the perinatal period and discuss the critical time windows in ILC development [40]. Schneider et al. (2019) demonstrated through fate-mapping that the perinatal period is a critical window for the distribution of innate tissue-resident immune cells within developing organs and that “a majority of peripheral ILC2 pools were generated de novo during the postnatal window“ [41]. Ignacio A (2024) proposes that the establishment of ILCs and the tissue lymphoid niche during early development may have consequences much later in life [42].
4. Developmental impact on mucosal immunity: The first years of life represent a window of active intestinal immune development during which the mucosal immune system is learning to distinguish commensal microbiota (promoting tolerance) from pathogenic bacteria (requiring IL-8-mediated immune activation). Dysbiosis during this developmental period may durably reprogramme intestinal immune responses, with reduced IL-8 production representing a pathological adaptation to aberrant dysbiotic signals. The normalization of IL-8 in older children suggests that compensatory mechanisms or microbiota stabilization occurs by school-age years.

It is important to acknowledge that reduced stool IL-8 in ASD may not be exclusively attributable to dysbiosis. Dietary patterns (e.g., selective eating, low-fibre intake) could reduce luminal microbial stimulation of IL-8 production independently of ASD diagnosis. Gastrointestinal symptom severity (altered motility, constipation) may affect cytokine concentrations via transit time effects. Future studies should systematically record dietary intake, stool consistency (Bristol Stool Scale), and gastrointestinal symptom severity to disentangle these potential confounders from dysbiosis-driven immune dysregulation.

### 4.3. IL-4 and Dysregulated Th2/Treg Responses in the Gut

IL-4 is a quintessential Th2 cytokine produced by CD4+ T cells, mast cells, and basophils. In the context of gut immunity, IL-4 plays several critical non-redundant roles:

1. IgA class-switching: IL-4, working in concert with TGF-β, promotes IgA class-switching by B cells in Peyer’s patches and gut-associated lymphoid tissue, a process dependent on activation-induced cytidine deaminase (AID) [43,44].
2. Treg differentiation: IL-4 signals, particularly in the context of commensal-derived aryl hydrocarbon receptor (AhR) ligands, promote the differentiation of naive CD4+ T cells toward Foxp3+ regulatory T cells (Tregs), a process critical for establishing immune tolerance [27].
3. Alternative macrophage activation: IL-4 directs macrophages toward an alternatively activated (M2) phenotype, promoting the resolution of inflammation and epithelial repair.

The mechanistic interpretations offered below are speculative hypotheses consistent with the present observations but not directly tested in this study. Each proposed mechanism requires experimental validation. The significant reduction in stool IL-4 in ASD children, particularly in severe ASD, may reflect dysregulated Th2/Treg development and impaired intestinal tolerance mechanisms: (i) Impaired Th2 polarization: Dysbiotic microbiota depleted of bacteria that normally provide Th2-polarizing signals (primarily segmented filamentous bacteria [SFB]) may fail to adequately stimulate Th2 responses and IL-4 production from gut-associated CD4+ T cells. We note that Faecalibacterium prausnitzii, which we had cited in an earlier draft, is more accurately characterized as a Treg-inducing and anti-inflammatory bacterium acting through butyrate-mediated IL-10 and Foxp3+ Treg induction, rather than through classical Th2 pathway activation. This correction has been made in the text. The reference to Faecalibacterium has been removed from this context [45]. (ii) Reduced Treg differentiation: Decreased stool IL-4 may reflect impaired Treg cell development, a consequence of both reduced IL-4 availability and dysbiosis-driven loss of AhR ligand-producing commensals. This would be predicted to compromise immune tolerance and permit dysbiosis-driven inflammation [46]. (iii) Decreased IgA production: Previous studies have documented reduced fecal IgA levels in ASD children [47,48,49]. The present finding of reduced stool IL-4 may reflect altered IgA-mediated mucosal immune regulation, although this was not directly assessed in the present study. This interpretation is speculative in the context of the present study as no IgA measurements were performed in this cohort. This represents a hypothesis that warrants direct testing in future studies through simultaneous measurement of stool IL-4 and secretory IgA. Previous reports of reduced fecal IgA levels in ASD children provide indirect support for this mechanistic hypothesis [47]. IgA is critical for immune exclusion (preventing pathogenic bacteria colonization through enhanced clearance) and for shaping the microbiota toward a health-promoting composition.

### 4.4. IL-1β: Age-Dependent Dysregulation with Developmental Reversal

The striking age-dependent pattern for IL-1β—significant reduction in younger children but normalization (and even trend toward elevation) in older ASD children—provides important mechanistic insight into developmental immune maturation in ASD.

IL-1β is a cardinal pro-inflammatory cytokine produced by macrophages and epithelial cells in response to microbial pathogen-associated molecular patterns (PAMPs) and damage-associated molecular patterns (DAMPs). While elevated IL-1β is typically associated with inflammation, paradoxically low IL-1β in the gut has been associated with impaired antimicrobial responses and altered epithelial barrier function [50,51,52].

The following developmental model for IL-1β represents a hypothesis generated from our cross-sectional observations and requires validation in longitudinal studies or mechanistic experiments. The reduction in IL-1β in younger ASD children may reflect the following: (i) Impaired innate immune priming: Dysbiosis may reduce the diversity and abundance of IL-1β-stimulating bacterial PAMPs—particularly the microbe-associated molecular patterns (MAMPs) from commensal microbiota that normally calibrate basal mucosal IL-1β production. We note that the alternative explanation of butyrate-mediated IL-1β suppression is inconsistent with ASD-associated dysbiosis literature, which documents the depletion of butyrate-producing bacteria (e.g., Faecalibacterium, Roseburia, Blautia) rather than enrichment. Therefore, reduced IL-1β in younger ASD children more likely reflects loss of commensal PAMP stimulation rather than active butyrate-mediated immune suppression. This revised interpretation has been incorporated in the manuscript. This mechanistic explanation remains a hypothesis requiring experimental validation. (ii) Developmental adaptation: In early childhood, dysbiosis-driven reduction in IL-1β may represent a pathological adaptation in which the intestinal immune system becomes refractory to dysbiotic bacterial stimulation. This could reflect an evolutionary trade-off in which the immune system reduces inflammatory responses (and associated local epithelial damage) at the cost of reduced antimicrobial defence.

The trend toward elevated (rather than merely normalized) IL-1β in older ASD children compared to older controls (median 0.29 vs. 0.18 ng/L, non-significant) admits at least two interpretations. First, it may reflect compensatory immune maturation as intestinal lymphoid tissues and microbiota–immune cross-talk stabilizes by late childhood. Second, an equally plausible explanation is that the observed increase reflects the early stages of chronic low-grade intestinal inflammation in older ASD children, consistent with the elevated pro/anti-inflammatory ratio observed in this age group and consistent with evidence that persistent dysbiosis leads to progressive, low-grade mucosal immune activation over time. We cannot distinguish these alternatives from the present cross-sectional data; longitudinal studies with repeated immune profiling from early childhood through adolescence are required to resolve this question.

### 4.5. Pro/Anti-Inflammatory Imbalance as an Integrated Biomarker

While individual cytokine reductions (IL-8, IL-4) were observed, the pro/anti-inflammatory ratio may provide integrated insight into immune balance. The 2.3-fold elevation in the median pro/anti ratio in severe ASD compared to controls (491 vs. 209) did not reach conventional statistical significance (*p* = 0.059, Mann–Whitney U) and should therefore be interpreted as a trend requiring replication rather than a confirmed finding. This non-significant trend may reflect a true biological shift toward pro-inflammatory immune balance in severe ASD, or may represent a chance finding given the large within-group variability (SD > 600 for all groups). Any conclusions regarding a pro-inflammatory immune phenotype in severe ASD must be treated as hypothesis-generating and should not be used to justify clinical decisions pending independent validation in larger cohorts.

The heterogeneity in pro/anti ratios within the ASD population (35% with ratios > 500, 40% with balanced ratios 100–500, 25% with anti-inflammatory ratios < 100) may indicate multiple distinct immune phenotypes within autism, potentially reflecting different biological pathways contributing to the ASD behavioural phenotype. This immune phenotyping may help explain differential responses to immune-modulating interventions: pro-inflammatory ASD children may benefit from anti-inflammatory treatments, while those with anti-inflammatory profiles might not. However, this hypothesis requires validation in prospective interventional studies.

### 4.6. Developmental Window for Dysbiosis–Immune Dysregulation in ASD

A major novel finding from this study is the restriction of key cytokine alterations (IL-8, IL-1β) to younger children (≤9.5 years), with normalization in older children. This developmental pattern has important biological and clinical implications:

Biological interpretation: The first decade of life represents a critical period for intestinal immune system development and microbiota maturation. During ages 0–3 years, the microbiota rapidly diversifies and establishes a relatively stable community structure [53,54]. During ages 3–10 years, this microbiota continues to refine and adapt to dietary and environmental changes. By late childhood/early adolescence (10+ years), microbiota composition and mucosal immune function have undergone substantial maturation relative to early childhood, although recent evidence indicates that both continue to be refined throughout adolescence and into early adulthood. We acknowledge that the term ‘adult-like stability’ in our earlier draft was an oversimplification; the current literature documents microbiota and immune changes continuing beyond age 10, as noted by Arrieta (2025) and Agace and McCoy (2017) [54,55]. The observed normalization of IL-8 and IL-1β differences in children > 9.5 years likely reflects the relative (rather than complete) stabilization of the relevant immunological parameters [56].

Dysbiosis during these developmental windows may have greater impact on mucosal immune development because the immune system is actively establishing tolerance mechanisms and learning to recognize commensal bacteria from pathogenic invaders. By adolescence, even in children with persistent dysbiosis, compensatory immune mechanisms (increased Treg function, improved barrier integrity, stabilized dysbiotic communities) may buffer against further immune dysregulation [55,57,58].

Clinical implications: These findings suggest that interventions targeting dysbiosis and immune dysregulation may be most effective when implemented in early childhood (ages 2–8 years), before immune compensatory mechanisms solidify. Microbiota-targeting therapies (probiotics, dietary prebiotics, microbial transplantation) implemented during this window may have greater efficacy in normalizing immune profiles and potentially ameliorating ASD symptoms than interventions in older children or adolescents.

Research implications: The age-dependent nature of these findings highlights the importance of developmental considerations in ASD research. Cross-sectional analyses combining all ages (as in prior studies) may obscure critical developmental effects. Future longitudinal studies tracking individual children’s microbiota and immune profiles across the developmental trajectory would clarify whether dysbiosis–immune dysregulation precedes or follows ASD symptom onset, and whether immune normalization is associated with improved behavioural outcomes.

### 4.7. Comparison with Prior Literature

Stool cytokine studies in ASD: Few prior studies have directly measured cytokines in stool from ASD children. A recent study of metataxomic and immunological analysis in Phelan–McDermid syndrome (a genetic ASD-associated condition) identified very low fecal IL-8 and IL-4 frequencies, consistent with the present findings [59]. The present study provides the first large-scale quantitative analysis of multiple fecal cytokines stratified by ASD severity and developmental age.

Blood cytokine studies in ASD: Meta-analyses and reviews have consistently documented elevated peripheral blood IL-1β, IL-6, IL-8, and TNF-α in ASD populations [8,9,34]. However, findings are heterogeneous, with some studies reporting no differences or even reduced markers in specific subgroups [9,60]. The present stool findings (reduced IL-8, IL-4) contrast with blood findings and suggest that compartmentalization of immune responses is important in ASD: systemic pro-inflammatory responses may coexist with local mucosal immune suppression.

This paradoxical compartmentalization could be explained by the “dysbiosis-driven translocation” model: dysbiosis-driven loss of barrier integrity permits bacterial translocation, triggering systemic (blood) pro-inflammatory responses while simultaneously reducing local mucosal (stool) immune activation [61,62].

Dysbiosis and microbiota studies: The dysbiotic microbiota composition previously documented in ASD (enriched Proteobacteria, depleted Bacteroidetes) directly aligns with the predicted effects on cytokine production observed in this study: Proteobacteria are LPS-rich Gram-negative bacteria that could trigger IL-8 responses if barrier integrity was preserved, but dysbiosis-associated barrier dysfunction may divert LPS responses systemically rather than locally [49,63].

### 4.8. Strengths

This study has several strengths that enhance the robustness and interpretability of our findings. First, the large sample size (N = 283) makes this one of the largest studies to date that directly measures stool cytokines in pediatric ASD and provides adequate power for group comparisons. Second, age-stratified and severity-stratified analyses revealed developmental and dose–response patterns that would have been obscured in aggregate analyses, offering novel mechanistic insight into ASD-related mucosal immune dysregulation. Third, we assessed a broad panel of nine cytokines spanning innate, Th1, Th2, Th17, and regulatory pathways, and complemented group comparisons with correlation and sensitivity analyses, thereby providing a comprehensive and methodologically transparent characterization of intestinal cytokine profiles in ASD.

### 4.9. Limitations

Several limitations should be considered when interpreting these results.

First, the cross-sectional design precludes causal inference regarding the temporal relationship between dysbiosis, mucosal cytokine changes, and ASD symptoms.

Second, stool cytokine measurements are influenced by several pre-analytical factors that were not systematically captured in this study. Intestinal transit time and stool consistency may substantially affect measured cytokine concentrations, with prolonged transit potentially concentrating cytokines and rapid transit or diarrhea diluting them. We did not systematically record Bristol Stool Scale scores or intestinal transit time, and therefore cannot exclude the possibility that differences in stool characteristics contributed to observed group differences. In addition, stool contains abundant proteases capable of degrading cytokines ex vivo. Although all samples were processed using a standardized protocol including protease inhibitor supplementation and storage at −80 °C, intraluminal proteolytic degradation prior to sample collection remains an uncontrolled variable. Furthermore, stool cytokine concentrations were not normalized to stool protein content, dry weight, or other measures of sample composition. Consequently, stool cytokine concentrations should be interpreted as relative indicators of intestinal immune activity rather than absolute measures of mucosal cytokine production.

Third, important potential confounders, including dietary patterns, food allergies, medication use beyond the predefined exclusion criteria, and gastrointestinal symptom severity were not systematically recorded. Dietary habits are known to substantially influence gut microbiota composition and intestinal immune function, and children with ASD frequently exhibit selective eating behaviours and food refusal. Similarly, medications such as antihistamines or anti-inflammatory agents could potentially influence stool cytokine concentrations. These uncontrolled factors may have contributed to the observed cytokine differences and should be systematically assessed in future studies.

In addition, we did not include parallel microbiome, metabolomic, or dietary assessments, which limits our ability to link specific microbial communities or metabolites to the observed cytokine phenotypes.

A further limitation is that cytokine measurements were based on single stool samples per participant, so we could not assess the intra-individual variability or temporal stability of mucosal immune profiles.

In addition, the marked sex imbalance between ASD and control groups may have influenced cytokine profiles despite additional sex-stratified analyses, and residual confounding cannot be excluded.

Moreover, multiple cytokines and subgroup comparisons were analyzed in an exploratory framework without formal adjustment for multiple testing; therefore, especially borderline *p*-values should be interpreted cautiously and validated in independent cohorts. Consequently, statistically significant findings should be regarded as hypothesis-generating and require replication in independent cohorts and longitudinal studies.

Finally, data completeness differed across cytokines due to assay sensitivity and values below the LOQ were excluded rather than modelled, which may introduce bias if low concentrations were differentially distributed between groups.

An additional limitation relates to the developmental threshold analysis. The age split at the population median (9.5 years) was empirically chosen and not biologically pre-specified. While multivariable regression models treating age as a continuous variable with an ASD × age interaction term confirmed a sex effect (β = −0.13, *p* = 0.016 for male sex on log-IL-8) and an age trend (β = 0.011 per year, *p* = 0.152), the ASD × age interaction itself did not reach statistical significance (β = 0.008, *p* = 0.498 for IL-8), suggesting the developmental effect may be threshold-like rather than linear. The stratified approach should therefore be considered exploratory and hypothesis-generating, pending longitudinal validation.

## 5. Conclusions

This large cross-sectional study of stool cytokine profiles in 283 children revealed significantly decreased IL-8 and IL-4 concentrations in children with ASD compared to typically developing controls. Critically, age-stratified analysis revealed that these immune alterations are developmentally restricted to early childhood (≤9.5 years), with normalization in older children, suggesting a potential developmental window when dysbiosis-driven immune dysregulation may have a greater impact on mucosal immunity. The pro/anti-inflammatory ratio was elevated in severe ASD, approaching statistical significance and suggesting immune balance dysregulation in the most behaviourally affected children.

These findings represent novel contributions to understanding the intestinal immune basis of ASD and are consistent with a dysbiosis-driven model in which altered microbiota composition during early childhood developmental windows disrupts the establishment of normal mucosal immune tolerance, leading to both local immune dysregulation (reduced IL-8, IL-4) and potentially paradoxical systemic immune activation (via bacterial translocation).

Clinical and research implications: (I) Stool IL-8 demonstrates a consistent, FDR-corrected association with ASD diagnosis (FDR-adjusted *p* = 0.037) that warrants further investigation as a candidate non-invasive biomarker. However, the sensitivity and specificity of stool IL-8 for ASD diagnosis have not been evaluated in this study, and clinical diagnostic utility should not be assumed from group-level comparisons. Validation in independent prospective cohorts with adequate clinical characterization is required before any clinical application is considered. (II) The age-stratified analysis suggests that cytokine differences between ASD and control children are most pronounced in children ≤ 9.5 years, which is consistent with the hypothesis that early childhood represents a critical period for dysbiosis–immune interactions in ASD, although the present findings do not provide direct evidence for this hypothesis. This cross-sectional observation does not demonstrate causality or establish a therapeutic window, and should not be interpreted as evidence that early intervention is effective. Longitudinal studies tracking immune and microbiota profiles from infancy are required to establish temporal relationships. (III) Multiple immune phenotypes appear to exist within autism (pro-inflammatory, balanced, anti-inflammatory), suggested by the heterogeneous pro/anti ratios; however, this observation is based on a non-significant trend (*p* = 0.059) and requires independent replication before clinical significance can be attributed. Immune phenotyping could potentially predict differential responses to immune-modulating interventions and guide precision medicine approaches to ASD treatment. (IV) Future research should integrate microbiota, immune, and behavioural profiling in longitudinal studies to establish temporal relationships and test causality. Multi-omic approaches combining 16S rRNA sequencing, metabolomics (SCFA measurement), and expanded immunological characterization would provide comprehensive mechanistic insight. (V) Compartment-specific immune profiling is essential in ASD research, with simultaneous measurement of mucosal (stool) and systemic (blood) immune markers to resolve paradoxes in the prior literature and understand the relationship between local and systemic immune dysregulation.

## Figures and Tables

**Table 1:** Demographic characteristics of study participants.

Characteristic	Control (N = 109)	Mild ASD (N = 104)	Severe ASD (N = 70)
N	109	104	70
Age (years), mean ± SD	10.6 ± 4.9	9.4 ± 4.2	9.6 ± 4.0
Age range (min–max)	0.9–21.5	1.9–18.2	3.5–19.5
Sex, male/female, N (%)	58/50 (53/47%)	82/22 (79/21%)	56/14 (80/20%)
CARS score, mean ± SD	16.9 ± 3.0	29.7 ± 5.0	43.9 ± 5.8
CARS range (min–max)	15–28	18–36	38–58

Data are presented as mean ± standard deviation (SD) for continuous variables and *n* (%) for categorical variables. CARS, Childhood Autism Rating Scale. *p*-values were obtained using one-way ANOVA for age and CARS scores and chi-square test for sex.

**Table 2:** Stool cytokine concentrations (ng/L)—in controls and children with ASD.

Cytokine	Control N	Control Median (Min–Max)	All ASD N	All ASD Median (Min–Max)	Mild ASD Median	Severe ASD Median	*p*-Value *	FDR-Adjusted *p*-Value (BH)	Significant
IFN-γ	87	3.83 (0.27–21.14)	136	4.32 (0.61–54.52)	6.16	2.73	0.4043	0.567	
IL-1α	109	157.12 (4.63–455.38)	172	135.64 (7.08–1141.17)	123.14	162.33	0.7205	0.721	
IL-1β	97	0.19 (0.00–45.39)	130	0.14 (0.01–6.79)	0.17	0.12	0.0904	0.203	
IL-4	63	0.30 (0.02–2.02)	114	0.28 (0.02–1.92)	0.28	0.24	0.0316	0.142	*
IL-6	99	0.57 (0.11–2.71)	148	0.68 (0.07–1.70)	0.73	0.66	0.0611	0.183	
IL-8	95	0.49 (0.01–35.28)	134	0.36 (0.04–12.78)	0.43	0.29	0.0041	0.037	**
IL-10	59	0.96 (0.05–26.97)	104	1.00 (0.06–20.00)	1.44	0.61	0.5038	0.567	
IL-17	105	1.94 (0.07–35.19)	170	2.18 (0.27–39.30)	2.19	1.84	0.1125	0.203	
TNF-α	91	8.77 (0.01–484.89)	138	9.35 (0.01–409.55)	10.48	7.91	0.4749	0.567	

Data are presented as median (min–max). Younger group: ≤9.5 years; older group: >9.5 years. *p*-values were obtained using the Mann–Whitney U test comparing ASD versus controls within each age stratum. * *p* < 0.05; ** *p* < 0.01.

**Table 3:** Age-stratified stool IL-8 concentrations (≤9.5 vs. >9.5 years).

Age Group	Control Median	ASD Median	*p*-Value	Effect
Younger (≤9.5 years)	0.56 ng/L	0.34 ng/L	0.0025	✓✓ Highly significant
Older (>9.5 years)	0.43 ng/L	0.42 ng/L	0.333	✗ Not significant

Data are presented as median (ng/L). Younger group: ≤9.5 years; older group: >9.5 years. *p*-values were obtained using the Mann–Whitney U test comparing ASD versus controls within each age stratum. ✓✓ indicates highly significant differences.

**Table 4:** Age-stratified stool IL-1β concentrations (≤9.5 vs. >9.5 years).

Age Group	Control Median	ASD Median	*p*-Value	Effect
Younger (≤9.5 years, N = 104)	0.21 ng/L	0.13 ng/L	0.0377	✓ Significant
Older (>9.5 years, N = 123)	0.18 ng/L	0.29 ng/L	0.621	✗ Not significant (reversed)

Data are presented as median (ng/L). Younger group: ≤9.5 years; older group: >9.5 years. *p*-values were obtained using the Mann–Whitney U test comparing ASD versus controls within each age stratum. ✓ indicates significant differences.

**Table 5:** Age-stratified stool IL-4 concentrations (≤9.5 vs. >9.5 years).

Age Group	Control Median	ASD Median	*p*-Value
Younger (≤9.5 years)	0.26 ng/L	0.24 ng/L	0.468
Older (>9.5 years)	0.35 ng/L	0.31 ng/L	0.105

Data are presented as median (ng/L). Younger group: ≤9.5 years; older group: >9.5 years. *p*-values were obtained using the Mann–Whitney U test comparing ASD versus controls within each age stratum.

**Table 6:** Age-stratified stool cytokine concentrations (≤9.5 vs. >9.5 years) for key markers.

Cytokine	Age Group	Control N	Control Median	ASD N	ASD Median	*p*-Value	FDR-Adjusted *p*-Value (BH)	Significant	Effect
IL-8	Younger (≤9.5 y)	40	0.560	76	0.340	0.0025	0.037	**	↓↓ STRONG
	Older (>9.5 y)	54	0.430	58	0.420	0.3408			Not significant
IL-4	Younger (≤9.5 y)	28	0.260	66	0.240	0.4680	0.142		Not significant
	Older (>9.5 y)	34	0.350	48	0.310	0.0832		~	Trend only
IL-1β	Younger (≤9.5 y)	38	0.210	66	0.130	0.0377	0.203	*	↓ REDUCED
	Older (>9.5 y)	58	0.180	64	0.290	0.6758			REVERSED
IFN-γ	Younger (≤9.5 y)	42	3.910	78	4.310	0.9407	0.567		Not significant
	Older (>9.5 y)	44	3.705	58	4.330	0.2928			Not significant

Data are presented as median (ng/L). Younger group: ≤9.5 years; older group: >9.5 years. *p*-values were obtained using the Mann–Whitney U test comparing ASD versus controls within each age stratum. * *p* < 0.05; ** *p* < 0.01; ~ *p* < 0.10 (trend). “Effect” qualitatively indicates direction and strength of the difference. The downward arrow ↓↓ indicates a strong decrease, whereas ↓ indicates lower cytokine concentrations in the ASD group compared with controls.

**Table 7:** Pro-/anti-inflammatory cytokine ratios by diagnostic group.

Group	N	Mean Ratio	Median Ratio	Range
Control	83	466 ± 603	209	1.7–2687
Mild ASD	94	538 ± 940	184	8.2–5051
Severe ASD	56	980 ± 1381	491	4.2–6534

Data are presented as mean ± standard deviation (SD), median, and range. Pro-/anti-inflammatory ratio = (IFN-γ + IL-1α + IL-1β + IL-6 + IL-8 + IL-17 + TNF-α)/(IL-4 + IL-10). Ratios are unitless.

**Table 8:** Pro-/anti-inflammatory cytokine ratio distribution by diagnostic group.

Group	N	Mean ± SD	Median	Min–Max	Q1–Q3	Elevation vs. Control
Control	83	466 ± 603	209	2–2687	97–575	—
Mild ASD	94	538 ± 940	184	8–5051	78–526	1.2×
Severe ASD	56	980 ± 1381	491	4–6534	101–1096	2.3× (*p* = 0.059)

Data are presented as mean ± SD, median, minimum–maximum (Min–Max), and interquartile range (Q1–Q3). Pro-/anti-inflammatory ratio = (IFN-γ + IL-1α + IL-1β + IL-6 + IL-8 + IL-17 + TNF-α)/(IL-4 + IL-10). “Elevation vs. Control” indicates fold-change in median ratio compared with controls; *p*-values were obtained using the Mann–Whitney U test comparing ASD subgroups versus controls..

## Data Availability

The datasets generated and analyzed during the current study are available from the corresponding author on request, in anonymized form and in accordance with institutional and ethical regulations.

## Supplementary Materials

The following supporting information can be downloaded at https://www.mdpi.com/article/10.3390/biomedicines14071559/s1, Table S1: Multivariable regression models for primary cytokines with continuous age and age × diagnosis interaction term.

## Abbreviations

The following abbreviations are used in this manuscript:

ASD	Autism spectrum disorder
AID	Activation-induced cytidine deaminase
AhR	Aryl hydrocarbon receptor
ANOVA	Analysis of variance
CARS	Childhood Autism Rating Scale
CI	Confidence interval
DSM-5	Diagnostic and Statistical Manual of Mental Disorders, Fifth Edition
DAMP	Damage-associated molecular pattern
GALT	Gut-associated lymphoid tissue
IBD	Inflammatory bowel disease
IFN-γ	Interferon-gamma
IL	Interleukin
ILC	Innate lymphoid cell
LOQ	Limit of quantification
LPS	Lipopolysaccharide
MAMP	Microbe-associated molecular pattern
PAMP	Pathogen-associated molecular pattern
PBS	Phosphate-buffered saline
SCFA	Short-chain fatty acid
SD	Standard deviation
TLR	Toll-like receptor
TNF-α	Tumour necrosis factor-alpha
Treg	Regulatory T cell
UMCL	University Medical Centre Ljubljana
