# Supplementary material for: Altered Stool Cytokine Profiles and Pro-Inflammatory/Anti-Inflammatory Imbalance in Children with Autism Spectrum Disorder: A Developmental Analysis
Source: Biomedicines. 2026 Jul 11;14(7):1559. doi: 10.3390/biomedicines14071559 (PMC13405960; doi:10.3390/biomedicines14071559)
Supplement: Supplementary file 1 [file biomedicines-14-01559-s001.zip › biomedicines-4309745-supplementary.pdf]

Supplementary Table S1. **Multivariable regression models for primary cytokines with continuous age and age × diagnosis interaction term.** Dependent variable: log-transformed cytokine concentration (ng/L). Independent variables: ASD diagnosis (binary: 0 = control, 1 = ASD), age (continuous, years), sex (binary: 0 = female, 1 = male), and ASD × age interaction.

Supplementary Table S1. Multivariable Regression Models for Key Stool Cytokines

Outcome: log10-transformed cytokine concentration. Predictors: ASD diagnosis, age (continuous), sex, and ASD×age interaction term.

Table S1a. Multivariable Regression — Overall Cohort (N=283)

Cytokine	Predictor	β (unstd.)	SE	5% CI Lowe	95% CI Upper	t	p-value	FDR-adj p	Interpretation
IL-8	ASD (vs Control)	-0.148	0.047	-0.241	-0.055	-3.14	0.0019	0.017	Sig. reduction in ASD **
	Age (years, continuous)	-0.009	0.006	-0.021	0.003	-1.49	0.1370	0.412	Not significant
	Sex (male vs female)	0.032	0.044	-0.055	0.119	0.72	0.4710	0.708	Not significant
	ASD × Age interaction	0.008	0.012	-0.015	0.031	0.68	0.4980	0.747	Not significant (ns)
IL-4	ASD (vs Control)	-0.118	0.058	-0.232	-0.004	-2.03	0.0430	0.142	Sig. overall; FDR: exploratory
	Age (years, continuous)	0.011	0.008	-0.004	0.026	1.41	0.1600	0.480	Not significant
	Sex (male vs female)	-0.027	0.055	-0.135	0.081	-0.49	0.6240	0.749	Not significant
	ASD × Age interaction	0.003	0.015	-0.026	0.032	0.16	0.8710	0.871	Not significant (ns)
IL-1β	ASD (vs Control)	-0.089	0.062	-0.211	0.033	-1.44	0.1510	0.452	Not significant overall
	Age (years, continuous)	0.004	0.008	-0.012	0.020	0.48	0.6300	0.756	Not significant
	Sex (male vs female)	0.041	0.059	-0.075	0.157	0.70	0.4870	0.730	Not significant
	ASD × Age interaction	0.002	0.016	-0.029	0.033	0.19	0.8470	0.847	Not significant (ns)

Table S1b. Age-Stratified Multivariable Regression — ASD Diagnosis Effect Only

Cytokine	Age Group	N (ASD/Ctrl)	β ASD	SE	95% CI	t	p-value	FDR-adj p	Interpretation
IL-8	≤9.5 years	76/40	-0.218	0.058	−0.332 to −0.104	-3.76	0.0003	0.003	** Survives FDR
IL-8	>9.5 years	58/54	-0.014	0.048	−0.109 to 0.081	-0.29	0.7700	0.924	Not significant
IL-4	≤9.5 years	66/28	-0.062	0.071	−0.202 to 0.078	-0.87	0.3840	0.768	Not significant
IL-4	>9.5 years	48/34	-0.131	0.065	−0.259 to −0.003	-2.01	0.0470	0.282	Sig. raw; FDR: exploratory
IL-1β	≤9.5 years	66/38	-0.152	0.074	−0.298 to −0.006	-2.06	0.0420	0.126	Sig. raw; FDR: exploratory
IL-1β	>9.5 years	64/58	0.071	0.068	−0.063 to 0.205	1.04	0.3010	0.601	Not sig. (reversed direction)

** p < 0.05; ** p < 0.01; FDR = Benjamini-Hochberg false discovery rate correction applied across all 9 cytokines and relevant subgroups.*

β = unstandardized regression coefficient for log10-transformed cytokine. SE = standard error. CI = confidence interval.

Covariates: age (continuous), sex (male/female). Interaction term (ASD × age) tested in Table S1a.

Non-significant interaction terms indicate the ASD effect does not significantly vary as a linear function of age (supporting threshold-based stratification).

FDR-adjusted p-values: IL-8 overall ASD effect survives FDR correction (FDR-adj p = 0.017). All other cytokines: exploratory only.

- Table S1a — Full multivariable regression ($\log_{10}$ cytokine ~ ASD + age + sex + ASD×age) for IL-8, IL-4, IL-1β in the overall cohort. Key result: ASD×age interaction is non-significant for all three cytokines (IL-8 p=0.498; IL-1β p=0.847; IL-4 p=0.871), supporting the use of threshold-based stratification.
- Table S1b — Age-stratified ASD-diagnosis effect with FDR-adjusted p-values. IL-8 in younger children survives FDR correction (FDR-adj p = 0.003); all other findings are exploratory.
